# Supplementary material for: Fast and Noninvasive Hair Test for Preliminary Diagnosis of Mood Disorders
Source: Molecules. 2022 Aug 20;27(16):5318. doi: 10.3390/molecules27165318 (PMC9416516; doi:10.3390/molecules27165318)
Supplement: Supplementary file 1 [file molecules-27-05318-s001.zip › molecules-1815621-supplementary.pdf]

Table S1. Summary of information about sex, age, and hair colors in the control group.

ID	SEX*	AGE	HAIR COLOR	ID	SEX*	AGE	HAIR COLOR
**C1**	K	21	Brown	**C39**	K	21	Brown
**C2**	M	22	Black	**C40**	M	21	Brown
**C3**	K	20	Blonde	**C41**	M	39	Brown
**C4**	K	47	Black	**C42**	M	48	Gray
**C5**	K	19	Louis	**C43**	K	19	Blonde
**C6**	K	18	Blonde	**C44**	K	22	Brown
**C7**	K	20	Louis	**C45**	K	51	Blonde
**C8**	K	27	Louis	**C46**	K	49	Brown
**C9**	M	38	Brown	**C47**	K	23	Brown
**C10**	M	19	Brown	**C48**	K	21	Brown
**C11**	K	20	Brown	**C49**	K	19	Brown
**C12**	M	22	Blonde	**C50**	K	19	Brown
**C13**	M	22	Blonde	**C51**	K	18	Blonde
**C14**	K	21	Brown	**C52**	K	18	Blonde
**C15**	K	22	Louis	**C53**	K	20	Blonde
**C16**	K	21	Black	**C54**	K	34	Brown
**C17**	K	22	Brown	**C55**	K	22	Brown
**C18**	K	21	Louis	**C56**	K	48	Brown
**C19**	K	21	Louis	**C57**	K	22	Blonde
**C20**	K	21	Louis	**C58**	K	43	Blonde
**C21**	K	21	Blonde	**C59**	K	35	Blonde
**C22**	K	46	Gray	**C60**	M	27	Black
**C23**	K	52	Gray	**C61**	K	22	Blonde
**C24**	K	29	Louis	**C62**	K	30	Brown
**C25**	K	24	Louis	**C63**	K	45	Louis
**C26**	K	51	Redheads	**C64**	K	48	Blonde
**C27**	M	23	Brown	**C65**	K	25	Louis
**C28**	K	40	Blonde	**C66**	K	38	Blonde
**C29**	K	25	Louis	**C67**	K	51	Brown
**C30**	M	27	Louis	**C68**	K	59	Brown
**C31**	K	43	Blonde	**C69**	K	51	Redheads
**C32**	M	53	Gray	**C70**	K	56	Black
**C33**	K	21	Brown	**C71**	K	28	Blonde
**C34**	M	21	Blonde	**C72**	K	47	Brown
**C35**	K	18	Brown	**C73**	K	45	Louis
**C36**	M	51	Black	**C74**	K	46	Brown
**C37**	K	31	Brown	**C75**	K	23	Brown
**C38**	K	18	Brown				

**W- woman, M- man,*

Table S2. Summary of information about sex, age, used drugs, the dose, dosage, and hair colours, in the patients group.

ID	SEX*	AGE	DRUG**	DOSE [mg]	DOSAGE	HAIR COLOR
P1	W	69	TRA<br>VEN	150<br>75	0-0-1<br>1-0-0	Grey
P2	W	43	SER<br>TRA	50<br>150	0-0-1<br>2-0-0	Grey
P3	W	55	VEN	75	1-0-0	Black
P4	W	39	SER	50	0-0-1	Black
P5	W	59	VEN	150	1-0-0	Black
P6	M	56	DUL<br>TRA	60<br>150	1-0-0<br>0-0-1	Grey
P7	W	47	MIR<br>VEN	15<br>75	1-0-0<br>1-0-0	Black
P8	W	49	SER<br>TRA	100<br>150	1-0-0<br>0-0-1	Grey
P9	M	64	DUL	30	1-0-0	Grey
P10	M	64	SER	50	1-1-0	Grey
P11	M	53	SER	50	1-1-0	Grey
P12	M	61	DUL<br>QUET	60<br>25	1-0-0<br>1-0-1	Grey
P13	M	48	VEN	75	1-0-0	Black
P14	W	37	SER	50	1-0-0	Black
P15	W	45	TRA<br>VEN	75<br>75	0-0-1.33<br>2-0-0	Blonde
P16	M	61	MIR<br>QUET<br>VEN	30<br>75<br>150	0-0-1<br>1-0-0<br>1-0-0	Grey
P17	K	42	VEN	75	1-0-0	Black
P18	M	51	VEN	75	1-0-0	Grey
P19	W	58	PAR	20	1-0-0	Grey
P20	M	37	CIT<br>QUET	10<br>200	1-0-0<br>0-0-1	Blonde
P21	W	49	DUL<br>FLUX<br>TRA	30<br>10<br>150	1-0-0<br>1-0-1<br>0-0-1	Blonde
P22	W	80	MIR	30	1-0-1	Grey
P23	M	57	DUL	60	1-0-0	Brown
P24	W	42	SER<br>TRA	50<br>50	1-0-0<br>1-0-0	Brown
P25	W	51	SER	50	1-0-0	Blonde
P26	W	51	QUET	75	1-0-0	Blonde
P27	M	55	SER	50	2-0-0	Grey
P28	K	44	QUET	40	1-0-1	Blonde
P29	M	41	VEN	150	1-0-1	Black
P30	W	37	OLA<br>SER	5<br>100	0-0-1<br>1-0-0	Brown
P31	W	47	FLUX<br>TRA	10<br>150	2-0-0<br>0-0-1	Blonde

P32	W	66	SER	100	1-0-1	Grey
P33	W	30	SER	50	1-0-0	Brown
P34	K	48	OLA	5	1-0-1	Blonde
			DUL	60	1-0-1	
P35	W	50	DUL	60	1-0-1	Blonde
P36	W	29	AMI	10	1-0-1	Redheads
			LAM	100	1-0-2	
P37	W	25	SER	50	2-1-0	Black
P38	M	42	TRA	150	1-0-1	Grey
P39	M	29	DUL	0,03	0-0-1	Blonde
			LAM	0,05	1-0-1	
			TRA	150	1-0-1	
P40	W	55	OLA	5	1-0-2	Blonde
			SER	50	1-0.5-0	
			TRA	300	0-0-0.5	

*W- woman, M- man,

**TRA – Taradone; SER - Sertraline; OLA - Olanzapine; LAM - Lamotrygine; DUL - Duloxetine; AMI - Amitryptyline; VEN - Venlafaxine; FLUX – Fluoxetine; MIR – Mirtazapine; QUET – Quetiapine;
